# Event Encryption for Neuromorphic Vision Sensors: Framework, Algorithm, and Evaluation

**DOI:** 10.3390/s21134320

**Published:** 2021-06-24

**Authors:** Bowen Du, Weiqi Li, Zeju Wang, Manxin Xu, Tianchen Gao, Jiajie Li, Hongkai Wen

**Affiliations:** 1 Department of Computer Science, University of Warwick, Coventry CV4 7AL, UK; B.Du@Warwick.ac.uk; 2 School of Software Engineering, Tongji University, Shanghai 201804, China; 1853401@tongji.edu.cn (W.L.); 1751926@tongji.edu.cn (Z.W.); 1953474@tongji.edu.cn (M.X.); 1953068@tongji.edu.cn (T.G.); jiajie_li@alumni.tongji.edu.cn (J.L.)

**Keywords:** neuromorphic vision sensor, event camera, event encryption, privacy, security

## Abstract

Nowadays, our lives have benefited from various vision-based applications, such as video surveillance, human identification and aided driving. Unauthorized access to the vision-related data greatly threatens users’ privacy, and many encryption schemes have been proposed to secure images and videos in those conventional scenarios. Neuromorphic vision sensor (NVS) is a brand new kind of bio-inspired sensor that can generate a stream of impulse-like events rather than synchronized image frames, which reduces the sensor’s latency and broadens the applications in surveillance and identification. However, the privacy issue related to NVS remains a significant challenge. For example, some image reconstruction and human identification approaches may expose privacy-related information from NVS events. This work is the first to investigate the privacy of NVS. We firstly analyze the possible security attacks to NVS, including grayscale image reconstruction and privacy-related classification. We then propose a dedicated encryption framework for NVS, which incorporates a 2D chaotic mapping to scramble the positions of events and flip their polarities. In addition, an updating score has been designed for controlling the frequency of execution, which supports efficient encryption on different platforms. Finally, extensive experiments have demonstrated that the proposed encryption framework can effectively protect NVS events against grayscale image reconstruction and human identification, and meanwhile, achieve high efficiency on various platforms including resource-constrained devices.

## 1. Introduction

Nowadays, vision sensors have been widely deployed and playing important roles in a variety of computer-vision-based applications. Traditional RGB cameras have been widely applied in various scenarios, such as video surveillance, face recognition and aided driving. However, traditional sensors cannot be used to capture fast movement, and work unsatisfactorily in challenging lighting conditions. In the past decade, several new kinds of vision sensors have been invented to overcome these disadvantages, and one of them is neuromorphic vision sensors (NVS), which delivers fascinating features such as high temporal resolution, broad dynamic range, and low energy consumption. The characteristics of NVS have attracted increasing attentions from both academia and industry, yielding promising achievements in activity recognition [1], aided driving [2], localization [3], and anomaly detection [4].

It is certain that unprotected images and videos would threaten users’ privacy and security, and accordingly, there exist many mature approaches (such as [5,6,7,8,9,10]) in solving traditional image and video security issues. Unlike RGB cameras which produce pixel-based images, NVS outputs a stream of events, which captures luminous intensity changes at each pixel in a fine-grained manner. Prior work assumed that NVS is secure and privacy-preserving simply because it does not produce visible images, and directly applied NVS in privacy-related scenarios. For example, Samsung developed an NVS-based in-home monitoring solution named SmartThings Vision [11]. However, the occurrence of grayscale image reconstruction approaches [12,13,14] seriously threatens the security of NVS. These approaches can be used to generate visible images from the NVS output, and therefore all security issues related to traditional images and videos remain existing and applicable to NVS applications, which are worth revisiting and investigating.

To enhance the security of NVS events with affordable overhead in communication and storage, we analyze potential security risks, design a threat model dedicated for NVS data, and propose a novel encryption framework that can protect NVS data against image reconstruction and human identification attacks with efficient performance on various platforms. Major contributions of this study are summarized as below:

- To the best of our knowledge, this work is the first to investigate the privacy challenges of NVS. Based on existing datasets, algorithms and systems, we have proposed a novel privacy threat model dedicated for NVS data.
- We have proposed and designed an efficient encryption framework for NVS data, which employs 2D chaotic mapping and effectively protects NVS events against grayscale image reconstruction and human identification.
- Extensive experiments have demonstrated both effectiveness and high efficiency of the proposed framework on a wide range of platforms, including resource-constrained devices.

The rest of this paper is organized as follows. In Section 2, we review related studies on NVS applications and encryption schemes on traditional cameras. In Section 3, we analyze security issues related to NVS, and propose a dedicated threat model. In Section 4, we propose a novel encryption framework with in-depth elaborations. In Section 5, we evaluate the framework in terms of its security and efficiency, using different datasets on various devices. Finally, we conclude this study in Section 6.

## 2. Related Work

### 2.1. NVS-Based Applications in Private Scenarios

Taking advantage of NVS, some datasets, approaches and solutions have been proposed in privacy-related scenarios. Samsung designs SmartThings Vision [11], an NVS-based home monitoring vision sensor. It can detect unexpected intruders and alert fall by analysing the movements with a privacy-preserving approach. This is the first NVS-based commercial product which employs its characteristics in security domains.

Face and eye tracking are also sensitive tasks, which involve facial and iris information. A direct face detection approach has been designed in [15], which utilises random forest and chooses the histogram of oriented gradients as features. Besides, an eye-blink tracking algorithm combined with face detection has been proposed in [16]. It firstly captures the temporal signature of an eye blink and then detects the face by the recognized eyes. In [17], kernelised correlation filters have been employed for face detection, and an NVS-based face dataset was published. Furthermore, an NVS-based near-eye gaze tracking dataset was made available to the public. These algorithms and datasets extended the applications of NVS in private scenarios, but the security issues in these scenarios deserve further attentions, since the NVS outputs can be reconstructed to traditional images.

Besides facial and iris features, other biometrics can also be used for human identification. In [18], a human identification approach is directly drawn from the gait recognition in RGB videos. This approach involves five phases: visualization of the event stream, human figure detection, estimation of optical flow, human pose estimation, and gait recognition based on neural features. EV-Gait [19] collected several gait datasets using NVS and trains a convolutional neural network (CNN) to tackle the gait recognition problem. EV-Gait-GCN [20] applied a graph convolutional network (GCN) to identify human by the gait. These algorithms can even utilize NVS outputs for recognition without the reconstruction of images or videos. As a countermeasure, encryption can effectively prevent recognizing objects and other unauthorized access, to protect the outputs in more general scenarios.

### 2.2. NVS Security Risks

NVS has shown its power to solve some privacy-related scenarios. However, some algorithms can be used to reconstruct high-resolution grayscale images from the stream of events, and it threatens the privacy of NVS-based applications. Given the requirement to apply existing vision algorithms to NVS, reconstruction has been accompanying NVS since its appearance. In [15], a patch-based dictionary is learnt from event streams offline, and reconstruction is executed online based on the dictionary. A variational model is proposed to estimate the behaviour of NVS, and the grayscale images are reconstructed from the model [21]. A self-supervised learning approach is proposed in [22], which combines optical flow and event-based photometric constancy.

In addition to grayscale image reconstruction, some video synthesis algorithms have been further proposed. E2VID [12] is an end-to-end neural network-based approach, which is trained with the data generated from the simulator. This approach shows a good generalization with real-world data. Generative Adversarial Networks (GANs) have been used to generate videos from event streams. Both conditional GAN [13] and enhanced Cycle-GAN [14] have shown their capabilities in generating high-quality videos.

Some works managed to generate super-resolution intensity images from events. EventSR [23] utilizes three neural networks to complete reconstruction, restoration and super-resolution tasks, respectively. Besides, another end-to-end neural network for super-resolution reconstruction is proposed in [24], which pairs the events and the optical flow to generate images.

These reconstruction and generation approaches have brought traditional vision algorithms into the domain of NVS, together with the security issues associated with traditional cameras. These approaches are treated as a means of attack for acquiring privacy-related visual information.

### 2.3. Encryption Algorithms for Visual Data

Privacy is a crucial challenge for all kinds of vision sensors. Prior to the presence of NVS, many encryption algorithms had been designed for streaming data, such as Data Encryption Standard (DES), International Data Encryption Algorithm (IDEA) and Advanced Encryption Standard (AES). However, these algorithms are inefficient for vision sensors, since visual data is redundant and the scale is large. Specific encryption schemes have been designed for various vision sensors.

The Arnold Cat Map is a basic algorithm for pixel scrambling in the space domain for encrypting images [5]. It applies matrix transformation to scramble adjacent pixels rapidly. Several encryption algorithms have extended Arnold Cat Map to achieve better performance. Furthermore, chaotic-system-based encryption algorithms employ the pseudorandom sequence to encrypt the values of pixels, such as Logistic Mapping [6] and Chebychev Mapping [7]. Besides, encryption schemes in the transform domain [25,26] and partial encryption schemes [27] are also used for image encryption. Nevertheless, these schemes cannot be directly applied for NVS, since their outputs are sparse in the space domain, which is different from images.

Video has the features of both streaming data and images, and thus video encryption should be designed with considerations on both security and efficiency. VEA [8] directly applies streaming data encryption, i.e., DES, to encrypt video stream. Compared with image encryption, this approach increases the efficiency of encryption while the security is preserved. CSC [28] utilizes three chaotic mapping algorithms to generate a chaotic sequence, and an XOR operation is involved in generating the ciphertext. Moreover, some video encryption algorithms take advantage of the video’s encoding format to encrypt the key information, such as the widely-used MSE [9] and MHT [10]. These video encryption schemes rely on the encryption of critical information in a single frame or adjacent frames. However, such an approach is not suitable for NVS either, because each NVS output holds a small amount of information only.

The point cloud is a new kind of visual data representing a 3D structure of an object. Some extended chaotic mapping algorithms [29,30] have been used to encrypt the point cloud. Furthermore, a series of random permutations and rotations have been employed to encrypt the point cloud by deforming the geometry [31]. The structure of the point cloud is three-dimensional, which is similar to that of NVS outputs. However, the minimum and maximum values of the encrypted point cloud in each dimension are not consistent with the original point cloud, which prevents the application of these encryption schemes for NVS.

## 3. NVS and Privacy Challenges

### 3.1. An Overview of NVS

The NVS, also known as the event camera, is a bio-inspired sensor that resembles human retina [32]. Traditional cameras produce visible images at a fixed rate, while NVS captures each pixel’s intensity changes asynchronously. Similar to the retina and the visual neural system, NVS outputs spatially sparse and asynchronous events instead of dense and asynchronous images. An event is produced whenever a pixel’s (logarithmic) intensity change exceeds a customized threshold at a particular time. When an event is triggered, the corresponding pixel memorizes the logarithmic intensity and monitors whether the change from the memorized value exceeds the threshold.

If an event is captured at a pixel, it will be transmitted from the pixel array to the periphery, where events are encoded to an event stream using address-event representation (AER) readout. The event stream can be read from a shared digital output bus, and the output rates, ranging from 2 MHz to 300 MHz, are higher than that of standard cameras. The output rates are related to the movement speed and the scene’s brightness changes, and thus NVS will generate more events in a second when moving faster. Furthermore, NVS supports high dynamic range (140 dB) with low power consumption.

### 3.2. Event Generation and Representation

**Figure 1:**
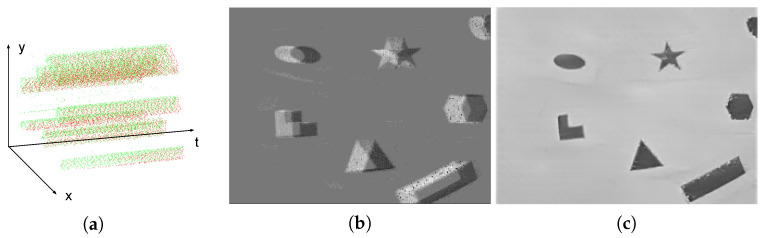
The different approaches to visualize a sample event stream. (**a**) Projecting events to a spatio-temporal space. (**b**) Calculating the number of events for each pixel in one second. (**c**) Reconstructing a grayscale image from a stream of events.

As aforementioned, NVS responds to the logarithmic intensity changes. The logarithmic intensity of a pixel is denoted as L(x,y,t), where *x* and *y* represent the position of the pixel and *t* is the timestamp. Whenever the change of the logarithmic intensity at a, i.e.,

(1) ΔL(x,y,t+Δt)=L(x,y,t+Δt)−L(x,y,t),

reaches the predefined threshold ±C (C>0), i.e.,

(2) ΔL(x,y,t+Δt)=pC,

a new event is generated. In the above formulas, Δt is the time elapsed since the last event occurred at the pixel, and p∈{−1,+1} represents the polarity of intensity change. p=+1 when the intensity change between the current event and the previous event at the same pixel is positive, while p=−1 when the change is negative. A generated event can be expressed as e=(t,x,y,p). Figure 1a illustrates a sample event stream in the duration of 0.1 s, while red and green points represent positive and negative events, respectively.

Considering that a single event contains little information, an event stream can always be represented as a frame along the time. A typical packing method is to sum up the polarity values of all events for each pixel in a time duration:

(3) $$E\left(x,y\right)=\sum_{t_{i}\in T}p_{i}\delta \left(x_{i}-x,y_{i}-y\right)$$

where $e_{i}=\left(t_{i},x_{i},y_{i},p_{i}\right)$ is an event occurred within the time duration *T*, and δ is the Delta function, which returns 1 at the origin or 0 otherwise. The packed result *E* is also known as an event image or an event frame and a sample is shown in Figure 1b.

### 3.3. Privacy Challenges and Threat Model

**Figure 2:**
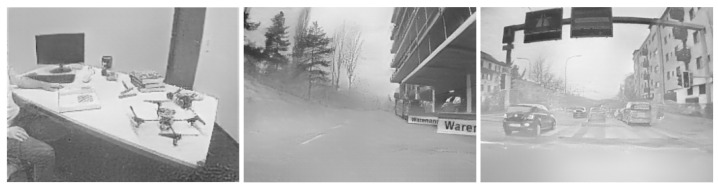
Reconstructed images from the DAVIS dataset [33] and DDD17 [34].

**Figure 3:**
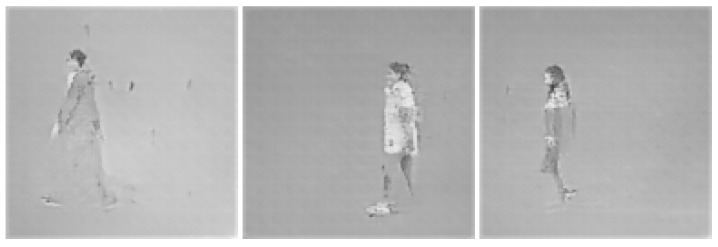
Failed reconstruction images based on the EV-Gait dataset [19].

Compared with a traditional RGB-based image, an event frame lacks detailed visible information, which drives its application in private scenarios such as in-home surveillance. However, some new algorithms can reconstruct grayscale frames from a series of events, threatening privacy-related applications. For example, a reconstructed image is shown in Figure 1c using the approach in [12], whose angle, edge and other details can be clearly recognized. More real-world reconstructed images are illustrated in Figure 2. These reconstruction algorithms are treated as a means of visualization attacks. However, such an attack may not be successful at all times. When the number of events is limited or no event appears in an area, the reconstructed images are blurry and cannot be recognized. Some examples of failed visualization attacks are illustrated in Figure 3. However, this does not imply that attackers cannot obtain any valuable information. NVS-based recognition algorithms commonly enable attackers to acquire desired information without image reconstruction. One example is a variety of human identification algorithms, which reaches up to 90% accuracy. Due to the privacy issues in identification tasks, these identification approaches are treated as another kind of attack, namely recognition attack. Based on aforementioned assumptions, the objectives, capabilities and knowledge of the threat model are defined as follows:

**Objectives:** the adversary wants to (i) reconstruct the visible images from the event stream (visualization attack) and/or (ii) identify different people (recognition attack) under the condition that no clear grayscale image can be reconstructed.
**Capabilities:** the adversary can access events during the transmission and storage processes. The adversary may not acquire the continuous integrated event stream, but a part of (maybe nonadjacent) events for attacking.
**Knowledge:** the adversary is supposed to know the encoding format of events (AER) and is able to use some public NVS-based algorithms to reconstruct images and identify different people.

Concretely, E2VID [12] is utilized to carry out the visualization attack, while EV-Gait (-IMG) [19] and EV-Gait-3DGraph [20] are used to perform the recognition attack. E2VID provides a well-trained end-to-end neural network to reconstruct images from a stream of events. It firstly packs the events in a short period to a 3D tensor and then co-operates with several previously reconstructed images to generate a new image following the recurrent UNet architecture. This neural network is trained on synthetic data from an event simulator and performs well on real data. Its well-trained model weights have been released, and are utilized for attacks and evaluation in this work. EV-Gait-IMG and EV-Gait-3DGraph can identify different people using event streams without reconstructing grey-scale images. EV-Gait-IMG packs events to an event frame, and a ResNet with a fully connected neural network is employed to extract features and identify different people. EV-Gait-3DGraph models a stream of events as a 3D graph and applies nonuniform OctreeGrid filtering for downsampling. A 3D graph is constructed according to the distance of *x*, *y* and *t*, and the GCN-based approach can work on the constructed graph for identification.

## 4. The Proposed Efficient Encryption Framework

**Figure 4:**
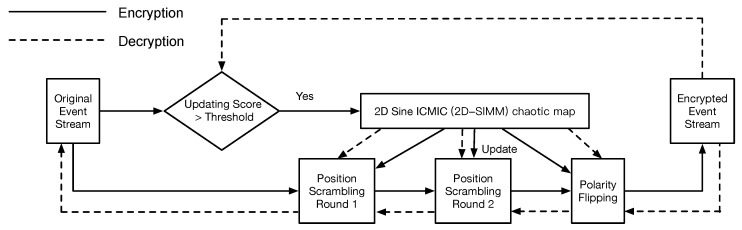
The flowchart of the proposed encryption and decryption framework. The updating score controls the updating speed of pseudo-random sequences, which are generated using chaotic mapping. The encryption (decryption) process consists of scrambling (restoring) positions and flipping (restoring) polarities.

Inspired by the encryption scheme for traditional images [35], we extend the approach of scrambling pixels’ positions for shuffling both events’ positions and their polarities. The encryption and decryption framework is illustrated as Figure 4. The main idea of the proposed framework is that several pseudo-random sequences, generated using a chaotic map and updated over a period of time, can securely map an event’s position and polarity to another position and polarity. Concretely, a two-dimensional chaotic map [35] firstly generates six pseudo-random sequences, which are employed to randomly shuffle original events (scrambling position and flipping polarity), and an indicator, updating score, is designed to control the updating speed of the sequences according to the type of NVS and the hardware configuration.

### 4.1. Pseudo-Random Sequence Generation

Chaotic systems are widely used techniques to generate pseudo-random sequences, which are applicable in image encryption. Due to its high sensitivity to initial conditions and parameters, chaotic systems can be employed to secure the generated pseudo-random sequence. For example, the sine map is a typical chaotic system, which is defined as:

(4) $$\begin{array}{cc} \; & x_{i+1}=\alpha \sin\left(x_{i}\right) \end{array}$$

where α is a control parameter, and the $x_{i+1}$ and $x_{i}$ should be ranged in [0,1]. Here, $x_{0}$, α and the iterations *i* jointly decide the current status of the chaotic system. However, a 1D chaotic system, such as a sine map, has few parameters and initial status, and the system status is relatively simple. To overcome such a disadvantage, high-dimensional chaotic maps have been proposed, but their computational complexity increases heavily compared with that of 1D chaotic systems. As a trade-off, two-dimensional (2D) chaotic maps can achieve better chaotic performance and introduce acceptable overhead.

A 2D chaotic map combines two different types of 1D chaotic maps. Besides the sine map, the iterative chaotic map with infinite collapse (ICMIC) [36] also shows robust chaotic characteristics, expressed as:

(5) $$\begin{array}{cc} \; & x_{i+1}=\sin\left(\frac{\beta }{x_{i}}\right) \end{array}$$

where $x_{i}\in \left(-1,1\right)$, $x_{0}\neq 0$ and β∈(0,+∞). β is also a control parameter. Based on these 1D chaotic maps, a two-dimensional Sine ICMIC modulation map (2D-SIMM) [35] is defined as:

(6) $$\left\{\begin{array}{c} x_{i+1}=a\sin\left(\pi y_{i}\right)\sin\left(\frac{b}{x_{i}}\right)\\ y_{i+1}=a\sin\left(\pi x_{i+1}\right)\sin\left(\frac{b}{y_{i}}\right) \end{array}\right.$$

where system parameters a,b∈(0,+∞). Here, *a*, *b*, $x_{0}$ and $y_{0}$ collectively describe the initial status of the chaotic system, and the iterations *i* decides the current status. *a* and *b* have been set as 1 and 5 respectively to generate pseudo-random sequences, because the system is a hyperchaotic map in this setting.

Since more than one pseudo-random sequences are required, to reduce the length of keys, the initial status of the system is decomposed to $x_{0}$ (52 bits), $y_{0}$ (52 bits), *H* (52 bits) and *G* (25 bits). The sequences share the same $x_{0}$, $y_{0}$ and *H*, but have different *G*. The initial status under condition *G*, $x_{0}^{G}$ and $y_{0}^{G}$ is donated as:

(7) $$\left\{\begin{array}{c} x_{0}^{G}=\left(x_{0}+GH\right)mod1\\ y_{0}^{G}=\left(y_{0}+GH\right)mod1 \end{array}\right.$$

Given that the width and height of the NVS resolution are *M* and *N*, we generate six pseudo-random sequences, whose lengths are *N*, *M*, *N*, *M*, 2N and 2M, respectively, and number these sequences from $r_{1}$ to $r_{6}$.

### 4.2. Encryption and Decryption Algorithms

The generated pseudo-random sequences are utilized to scramble the positions of events and flip the corresponding polarities. During the encryption process, there are two rounds for scrambling and one round for flipping. The former focuses on changing the positions of events, and the latter modifies the polarities with the position shuffle.

**Position Scrambling Round:** the first round of scrambling utilizes the sequence $r_{1}$ and $r_{2}$, while $r_{3}$ and $r_{4}$ are used for the second round. Here, $r_{1}^{i}$ denotes the *i*th element of the sequence $r_{1}$. The scrambling on *y* under $r_{1}$ is formulated as:

(8) $$f_{r_{1}}\left(x,y\right)=\left(x,\left(y+r_{1}^{x}\right)\;\mathrm{mod}\;M\right)$$

which means that an event at the position (x,y) moves $r_{1}^{x}$ steps right-forward to $\left(x,y+r_{1}^{x}\right)$. If the changed *y* exceeds the boundary *M*, the movement will start from the (x,0) and end with $\left(x,\left(y+r_{1}^{x}\right)\;\mathrm{mod}\;M)\right)$. Similarly, the scrambling on *x* under $r_{2}$ is formulated as:

(9) $$f_{r_{2}}\left(x,y\right)=\left(\left(x+r_{2}^{y}\right)\;\mathrm{mod}\;N,y\right)$$

The scrambling in the *x* direction is conducted after the *y* direction scrambling. After completing the first *x* and *y* direction scrambling, spatially adjacent events will be distributed to different positions. However, one round is not enough to scramble all events thoroughly [35]. Thus, the second round scrambling is executed again using the same methods but based on $r_{3}$ and $r_{4}$.

**Polarity Flipping Round:** after scrambling the positions of events, polarity flipping is associated with the scrambled position and the original polarity. There are two steps to flip the polarity, which shuttle the position as well. The first step is to flip the polarity based on the scrambled *y* and the original polarity, while the second step flips it according to the scrambled *y* and the first step’s result. Specifically, the $r_{5}$ is utilized for the first step, whose length is 2N. An event, which is processed after position scrambling round and is located at (x,y,p), is associated with the (p∗N+y)th element of the $r_{5}$, where x∈[0,M−1], y∈[0,N−1] and p∈0,1. Then, a sort operation S is conducted on the $r_{5}$, denoted as $S_{r_{5}}$. A new sequence $r_{5}^{\prime }$ is acquired, and the original element, located at p∗N+y, will be relocated at $l^{\prime }=S_{r_{5}}\left(p*N+y\right)$. $l^{\prime }$ is mapped to $\left(l^{\prime }\;\mathrm{mod}\;N,\left\lfloor \frac{l^{\prime }}{N}\right\rfloor \right)$. This transformation can be expressed as:

(10) $$f_{r_{5}}\left(x,y,p\right)=\left(x,S_{r_{5}}\left(p*N+y\right)\;\mathrm{mod}\;N,\left\lfloor \frac{S_{r_{5}}\left(p*N+y\right)}{N}\right\rfloor \right))$$

Similarly, the transformation of second step is conducted as:

(11) $$f_{r_{6}}\left(x,y,p\right)=\left(S_{r_{6}}\left(p*M+x\right)\;\mathrm{mod}\;M,y,\left\lfloor \frac{S_{r_{6}}\left(p*M+x\right)}{M}\right\rfloor \right))$$

**Figure 5:**
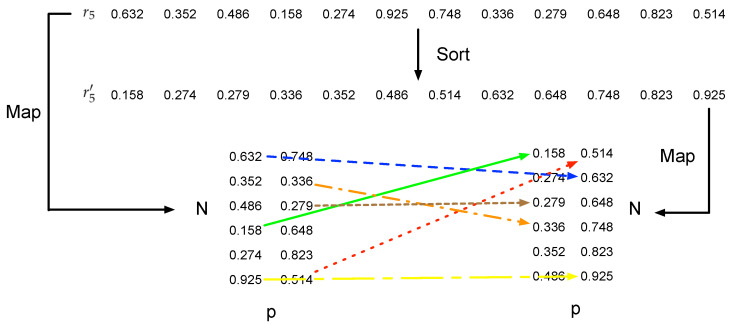
A polarity flipping example using $r_{5}$ to shuttle *y* and *p*.

An example of polarity flipping using $r_{5}$ is illustrated in Figure 5. In this example, (x,0,0) is mapped to (x,1,1), which is marked as the blue line, while the orange line shows that (x,1,1) is mapped to (x,3,0).

The encryption algorithm can be summarized in Algorithm 1. The position scrambling is from line 1 to line 4, while the polarity flipping begins from line 5.
**Algorithm 1:** Encryption Algorithm    **Input :** $r_{1},\ldots ,r_{6}$: pseudo-random sequences 
   
(t,x,y,p): an event 
   
M,N: the width and height of the NVS
    **Output:** $\left(t,x^{\prime },y^{\prime },p^{\prime }\right)$: an encrypted event1$y^{\prime }=\left(y+r_{1}^{x}\right)\;\mathrm{mod}\;N$;2$x^{\prime }=\left(x+r_{2}^{y^{\prime }}\right)\;\mathrm{mod}\;M$;3$y^{\prime }=\left(y+r_{3}^{x^{\prime }}\right)\;\mathrm{mod}\;N$;4$x^{\prime }=\left(x+r_{4}^{y^{\prime }}\right)\;\mathrm{mod}\;M$;5$r_{5}^{\prime }=Sort\left(r_{5}\right)$;6Generate $S_{r_{5}}$ which maps the index of each element in the original sequence of $r_{5}$ to the sorted $r_{5}^{\prime }$;7$p_{temp}=\left\lfloor \frac{S_{r_{5}}\left(p*N+y^{\prime }\right)}{N}\right\rfloor$;8$y^{\prime }=S_{r_{5}}\left(p*N+y^{\prime }\right)\;\mathrm{mod}\;N$;9$r_{6}^{\prime }=Sort\left(r_{6}\right)$;10Generate $S_{r_{6}}$ which maps the index of each element in the original sequence of $r_{6}$ to the sorted $r_{6}^{\prime }$;11$p^{\prime }=\left\lfloor \frac{S_{r_{6}}\left(p_{temp}*M+x^{\prime }\right)}{M}\right\rfloor$;12$x^{\prime }=S_{r_{6}}\left(p_{temp}*M+x^{\prime }\right)\;\mathrm{mod}\;M$;

The processes of encryption and decryption are symmetrical because the operations in encryption are reversible. The decryption begins with recovering the polarity, and then each pixel is restored to its corresponding location. Algorithm 2 presents the decryption process. Similar to encryption, the sorted sequences $r_{6}^{\prime }$ and $r_{5}^{\prime }$ are generated from the pseudo-random sequences $r_{6}$ and $r_{5}$. Given the indexes of the sorted sequences, the inverse map $S^{\prime }$ returns the indexes of the pseudo-random sequences. From line 1 to line 8, the randomly flipping polarity is recovered back to its original polarity. Line 12 to line 15 shows the inverse transformation of position scrambling.
**Algorithm 2:** Decryption Algorithm    **Input** : $r_{1},\ldots ,r_{6}$: pseudo-random sequences 
    $\left(t,x^{\prime },y^{\prime },p^{\prime }\right)$: an encrypted event 
    M,N: the width and height of the NVS
    **Output:** (t,x,y,p): an event1$r_{6}^{\prime }=Sort\left(r_{6}\right)$;2Generate $S_{r_{6}}^{\prime }$ which maps the index of each element in the sorted sequence $r_{6}^{\prime }$ to the original sequence $r_{6}$;3$p_{temp}=\left\lfloor \frac{S_{r_{6}}^{\prime }\left(p^{\prime }*M+x^{\prime }\right)}{M}\right\rfloor$;4$x=S_{r_{6}}\left(p^{\prime }*M+x^{\prime }\right)\;\mathrm{mod}\;M$;5$r_{5}^{\prime }=Sort\left(r_{5}\right)$;6Generate $S_{r_{5}}^{\prime }$ which maps the index of each element in the sorted sequence of $r_{5}^{\prime }$ to the original sequence $r_{5}$;7$p=\lfloor \frac{S_{r_{5}}^{\prime }\left(p_{temp}*N+y^{\prime }\right)}{N}\rfloor$;8$y=S_{r_{5}}\left(p_{temp}*N+y^{\prime }\right)\;\mathrm{mod}\;N$;9$x=(x-r_{4}^{y})\;\mathrm{mod}\;M$;10$y=(y-r_{3}^{x})\;\mathrm{mod}\;N$;11$x=(x-r_{2}^{y})\;\mathrm{mod}\;M$;12$y=(y-r_{1}^{x})\;\mathrm{mod}\;N$;

### 4.3. Pseudo-Random Sequence Updating

Although the encryption on the event stream prevents reconstructing grayscale images, events with the same position and polarity will be maped to another same position and polarity. If constant pseudo-random sequences are used from the beginning to the end, the adversary can attack by mapping the original event stream to the encrypted one before the transmission. It is therefore necessary to update the pseudo-random sequences frequently. However, high updating frequency will reduce the efficiency of encryption, and thus it is an important factor to decide when to update the sequences. Here, we define an *updating score* to decide whether to update the pseudo-random sequences when a new event occurs, according to the type of NVS and hardware configuration. Three parameters, the sensor’s resolution (N×M), the platform’s processing speed (*K*), and the number of processed events since the last update (*L*), are considered affecting the updating score. The relationship between these three values and the score is expressed as:

(12) $$\mathrm{Updating}\;\mathrm{Score}=\log_{10}\left(\frac{L}{N\times M}\times K\right)$$

The large resolution leads to more relational mappings between unencrypted events and encrypted events. When the number of events processed is fixed, higher resolution implies that more relational mappings have not been involved, and thus the level of security is relatively higher. Moreover, the higher processing speed of the platform enables more frequent updating. For example, compared with Raspberry Pi, the cloud server can perform updates more frequently to achieve higher security. Finally, the effect of *L* is intuitive: with the more events processed since the last update, it is more desirable to perform the update.

## 5. Evaluation

In order to conduct evaluations in several perspectives, three public NVS-based datasets were utilized. The first one was the DAVIS event camera dataset [33], a real-world dataset captured in various scenarios such as labs, offices and campuses. This dataset was published in 2017, which has been used for evaluations in a number of prior studies. DDD17 [34] is an end-to-end DAVIS driving dataset, which records NVS events in driving scenarios on a highway and in a city under different weather conditions. In this study, qualitative and quantitative evaluations for visualization attacks are conducted using these datasets, as well as efficiency analysis and secret key analysis. EV-Gait [19] published the NVS data collected for human identification by their gaits. Because the number of events for each record was small, the visualization attack on this dataset did not succeed. Therefore, this dataset was employed to evaluate all tasks except for visualization attack prevention.

Due to the lack of previous encryption schemes for NVS, we defined two kinds of partial encryption schemes, inspired by the keyframe encryption method on video [37], to evaluate the proposed encryption framework. The first baseline encryption schemes was to apply streaming data encryption, Advanced Encryption Standard (AES), directly to a part of events. Under this scheme, encrypted events and unencrypted events could be easily distinguished, and attackers could utilize unencrypted events to perform attacks. We called this encryption algorithm the partial discarding algorithm. The second baseline was to apply the scrambling method to a part of events, which meant that the unencrypted events could not be identified from all events. We named this algorithm as the partial scrambling algorithm.

### 5.1. Evaluation for Visualization Attacks

To perform the evaluation for visualization attacks, the grayscale images were firstly reconstructed from the original stream using E2VID [12]. After encrypting the event stream, the same configuration of E2VID was utilized to generate encrypted grayscale images.

#### 5.1.1. Qualitative Evaluation

**Figure 6:**
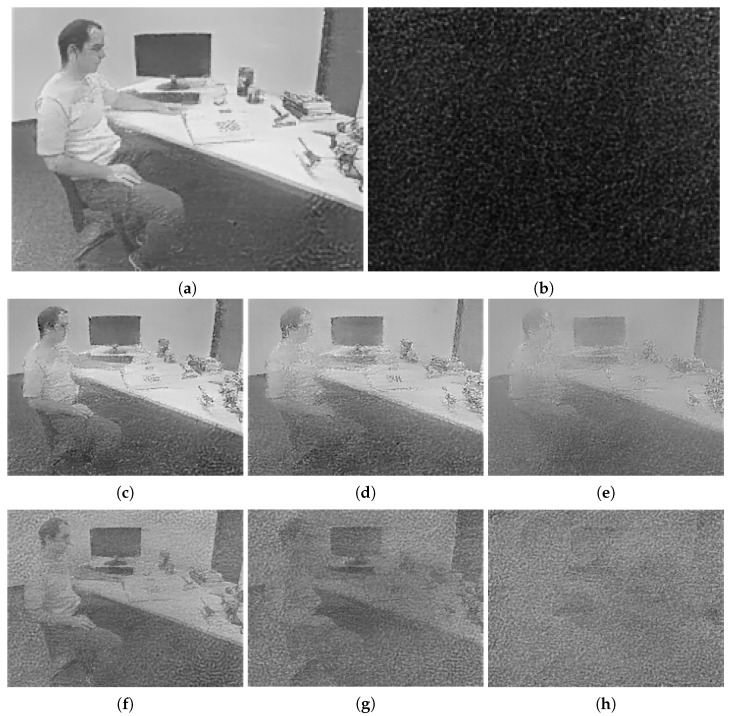
The reconstructed grayscale images of the same event stream under different conditions. (**a**) Image reconstructed from the original event stream. (**b**) Image reconstructed from encrypted events using our proposed framework. (**c**–**e**) Images reconstructed from 50%, 67%, and 75% encrypted events using the partial discarding algorithm, respectively. (**f**–**h**) Images reconstructed from 50%, 67%, and 75% encrypted events using the partial scrambling algorithm, respectively.

The reconstructed image produced from the original event stream is shown as Figure 6a, while the corresponding image produced from the encrypted event stream is illustrated as Figure 6b. Since the architecture of E2VID was recurrent, an error in the previous reconstruction was propagated to the next, and thus the generated image after encryption was nearly all dark. The reconstructed images corresponding to the partial discarding algorithm with different encryption percentage values are illustrated as Figure 6c–e. It can be observed that even when discarding 75% events, the outline of a person and a screen could still be recognized. The partial scrambling algorithm could not effectively prevent the visualization attacks, shown in Figure 6f–h, although it outperformed the partial discarding algorithm. Some shadows of the person and the screen could still be figured out. Compared with these two baselines, our proposed encryption algorithm achieved the best effectiveness.

**Figure 7:**
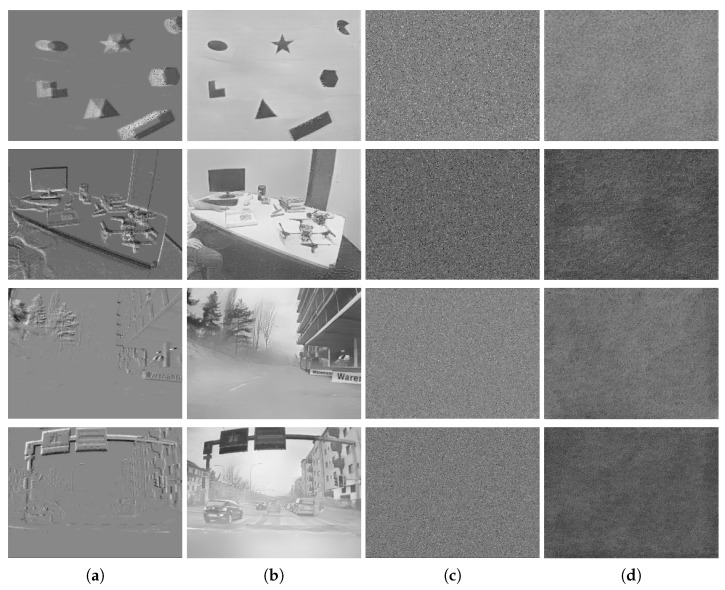
The qualitative evaluation of encryption. (**a**) The event images based on unencrypted events. (**b**) The reconstructed images based on unencrypted events. (**c**) The event images based on encrypted events using the proposed algorithm. (**d**) The reconstructed images based on encrypted events using the proposed algorithm.

Here, more event frames and reconstructed images before and after using the proposed encryption algorithm are shown in Figure 7. The first column presents the raw event frames, and the second column presents the reconstructed grayscale images produced by the unencrypted data. The third column shows the event frames after encryption, while the last column displays the reconstructed images after encryption. The first two rows present the results using the DAVIS dataset, while the last two rows demonstrate the results using DDD17. As shown in Figure 7, no outline or detail could be distinguished in the encrypted images (both event images and grayscale images), and the two images, before and after encryption, were totally different. It can therefore be concluded that the proposed encryption algorithm could be applied for diverse NVS hardware and scenarios.

#### 5.1.2. Quantitative Evaluation

In order to quantitatively evaluate the proposed encryption algorithm’s performance, we adopted Peak Signal to Noise Ratio (PSNR), Structural Similarity (SSIM), Unified Averaged Changed Intensity (UACI) and the Number of Pixel Changing Rate (NPCR) as metrics for comparing the images reconstructed from the original event stream and the encrypted one. Given the original reconstructed image *I* and the reconstructed image $I^{\prime }$ based on the encrypted event stream, the definitions of these metrics were as follows:

(13) $$PSNR=10\log_{10}\left(\frac{M\times N}{\sum_{n,m}^{n\leq N,m\leq M}{\left(I\left(n,m\right)-I^{\prime }\left(m,n\right)\right)}^{2}}\right)$$

(14) $$SSIM=\frac{\left(2\mu_{I}\mu_{I^{\prime }}+C_{1}\right)\left(2\sigma_{II^{\prime }}+C_{2}\right)}{\left(\mu_{I}^{2}+\mu_{I^{\prime }}^{2}+C_{1}\right)\left(\sigma_{I}^{2}+\sigma_{I^{\prime }}^{2}+C_{2}\right)}$$

(15) $$UACI=\frac{1}{M\times N}\sum_{n,m}^{n\leq N,m\leq M}\left(I\left(n,m\right)-I^{\prime }\left(m,n\right)\right)$$

(16) $$PNCR=\frac{1}{M\times N}\sum_{n,m}^{n\leq N,m\leq M}\delta \left(I\left(n,m\right)-I^{\prime }\left(m,n\right)\right)$$

The comparison was performed between the original reconstructed images and the processed images after the proposed encryption and the other two baselines, and the results are presented in Table 1 and Table 2. According to the results, the proposed encryption algorithm achieved the lowest values for PSNR and SSIM, and the highest for UACI and NPCR, compared with the baselines. The average PSNR value based on our algorithm was lower than 6.5, while that of other baselines exceeded 10.5. Our algorithm also achieved the lowest SSIM values in all sequences. The UACI and NPCR of the proposed algorithm were the best for most of the sequences, and slightly lower than that of the partial scrambling encryption algorithm. The PSNR between the original reconstructed image and the reconstructed image after encryption with the outdoor running sequence was higher than that with other sequences, because most of the pixels in the reconstructed images with the outdoor running sequence were gray, which implies that their intensity value was close to 0.5. Compared with scrambling 95% of events, the proposed encryption algorithm additionally flipped the polarity of events, making the encrypted events’ polarities distributed uniformly in {+1,−1}. Under this mechanism, the pixel values of the reconstructed image after encryption were also close to 0.5. Although our algorithm did not achieve the best performance on PSNR, UACI and NPCR using the outdoor running sequence, the uniformly distribution characteristic can effectively destroy the original pixel value distribution, leading to the best SSIM performance. Overall, our algorithm also outperformed others in the quantitative evaluation.

### 5.2. Evaluation for Recognition Attacks

**Figure 8:**
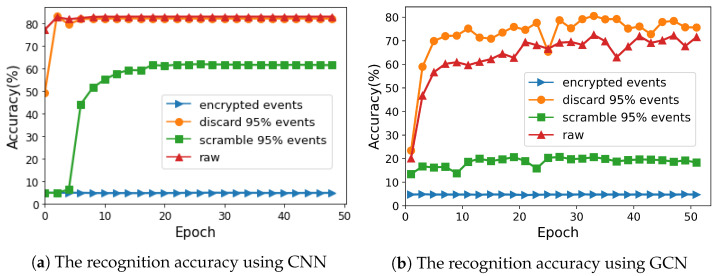
The accuracy of recognition attacks using event frame-based CNN and sparse event-based GCN under different conditions.

For the recognition attacks, the accuracy was employed as the metric to quantitatively validate how the encryption algorithm prevented the deep learning-based identification attacks, including an event frame-based CNN and a sparse event-based GCN. The accuracy values with different encryption algorithms at different epochs are presented in Figure 8.

Using the CNN approach, recognition attack could achieve 86% accuracy on original events and 81% accuracy on 95%-encrypted events using the partial discarding algorithm. Moreover, For 95%-encrypted events using the partial scrambling algorithm, the attack reached approximately 60% accuracy. In contrast, based on the our proposed algorithm, only 5% identification was correct, equal to the random guess. It is clear that the performance of our algorithm in recognition attack was as good as its performance in visualization attack, because the CNN-based attack extracted features from the packed frames, which were similar to the reconstructed images. In conclusion, the proposed algorithm effectively prevented the CNN-based attack and outperformed other encryption algorithms.

The performance under the GCN based-attack depended on the events that were used to constructed the 3D graph. The attack on the events using the partial discarding algorithm could achieve about 73% accuracy before the 50th epoch, while that using the partial scrambling algorithm could achieve about 20% accuracy. In contrast, with our proposed encryption algorithm, the attack achieved only 5% accuracy. This result shows that the proposed algorithm successfully destroyed the relationships among events in 3D spaces.

### 5.3. Secret Key Analysis

**Figure 9:**
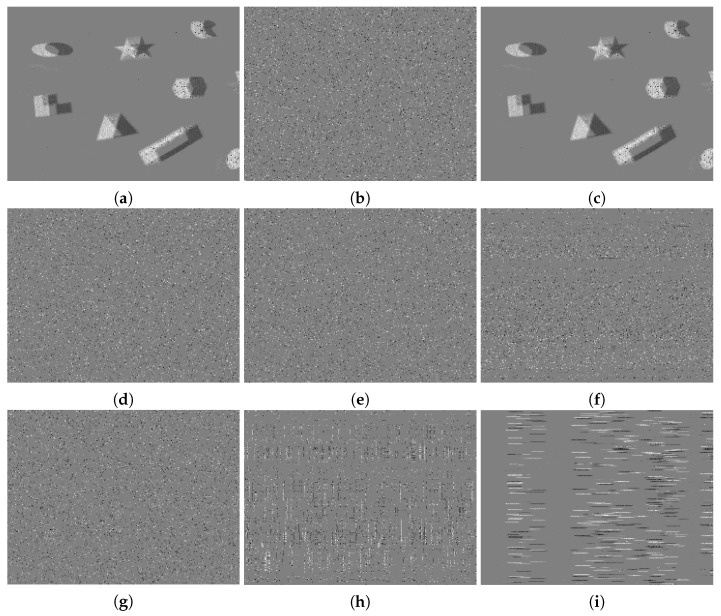
The sensitivity study on the secret keys. (**a**) The original event image. (**b**) The event image after encryption. (**c**) The event image after decryption with the correct secret key $K_{0}$. (**d**–**i**) The event images after decryption with incorrect secret keys $K_{1}$, $K_{2}$, $K_{3}$, $K_{4}$, $K_{5}$ and $K_{6}$, respectively.

The space of the secret key is an important factor affecting the application security in real scenarios. A large keyspace can make it difficult for attackers to enumerate the possible keys. The secret key, including $x_{0}$, $y_{0}$, *H* and *G*s, was 306 bits in length, and the keyspace was about $2^{306}$. It is impossible to crack the key by the brute-force attacks in a reasonable duration. Apart from the keyspace, the sensitivity of the encryption algorithm to the secret key also affected the usage. The original event frame and the decrypted event frames are illustrated in Figure 9. The correct key, $K_{0}$, could be used to decrypt the encrypted event frame. $K_{1}–K_{6}$ were the incorrect keys, which were produced by modifying the last bits of *G*s. The images produced by the decrypted events with slightly different keys, $K_{1}$ to $K_{6}$, were totally different from the event image using the right key. Therefore, the proposed encryption algorithm was sensitive enough to the secret key.

### 5.4. Efficiency Analysis

The proposed encryption was performed on different platforms for evaluation. For outdoor surveillance purposes, the real-time requirement on a resource-constrained platform is compulsory. The communication between the NVS platforms and the servers requires both encryption and decryption. These experiments were conducted on a Raspberry Pi, a desktop server and a cloud server. The Raspberry Pi ( 4b edition) was equipped with 8 GB RAM. The desktop server was equipped with an Intel Core i9-9900K@3.6 GHz processor with 32 GB RAM provided by Tongji University in Shanghai, China, and the cloud server was equipped with an Intel Xeon Platinum 8269CY@2.5 GHz and 64 GB RAM provided by Aliyun in Shanghai, China.

**Figure 10:**
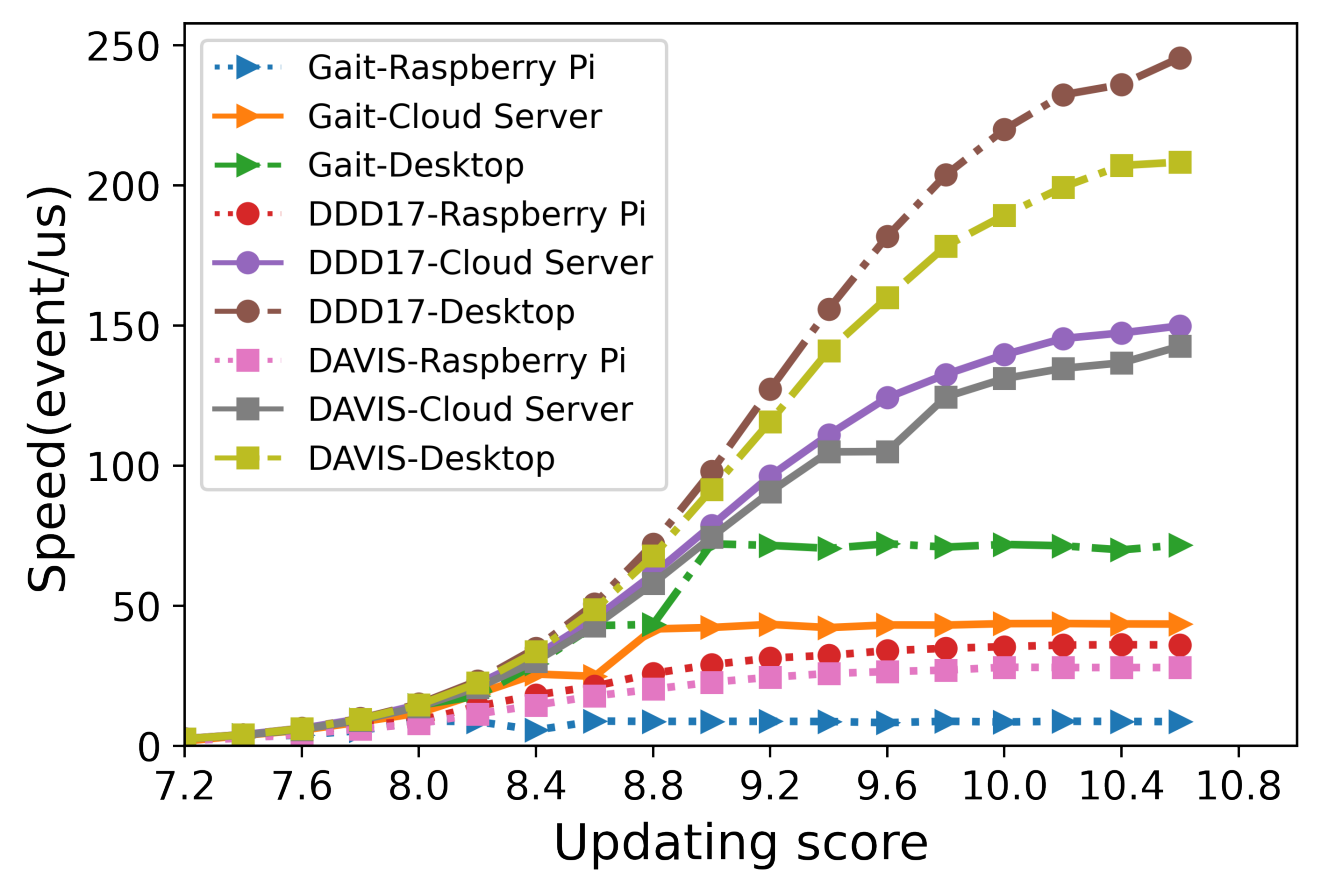
The relationship between the updating score and the number of events processed per second (in μs) using different NVSs on various platforms.

The time (μs) spent on encrypting each event is measured and listed in Table 3, Table 4 and Table 5. When the updating score was 7.2, the average time to encrypt one event was less than 0.60 μs on Raspberry Pi. When the updating score increased to 8.4, encrypting one event only consumed about 0.20 μs on Raspberry Pi, and only 0.04 μs on both desktop and cloud servers. The numbers of processed events per second with different updating scores are demonstrated in Figure 10. On the Raspberry Pi, more than tens of millions of events could be encrypted per second. These experimental results indicated that the proposed encryption framework worked efficiently on various platforms, including resource-constrained devices.

## 6. Conclusions

Neuromorphic vision sensor (NVS) is a new kind of bio-inspired sensor that can generate a stream of impulse-like events (rather than synchronized image frames generated by traditional RGB-based vision sensors), which greatly reduces the sensor’s latency and broadens its applications in surveillance and identification. However, the privacy issue related to NVS remains a significant challenge. To the best of our knowledge, this work is the first to investigate the privacy and security of NVS. Major contributions of this study are summarized as follows.

Firstly, we have identified and investigated major privacy issues related to NVS, and proposed a threat model that reveals serious security risks of NVS-based applications. Given the capabilities and knowledge defined in the model, adversaries can perform visualization and recognition attacks to achieve their objectives.

Secondly, we have proposed and designed an efficient encryption framework for protecting NVS data against visualization and recognition attacks. The framework incorporates a 2D chaotic mapping-based encryption algorithm and a secret key updating mechanism based on an updating score.

Finally, extensive experiments have been conducted, which demonstrated that the proposed framework can effectively and efficiently protect NVS events against grayscale image reconstruction and human identification. Specifically, the proposed encryption framework can be efficiently executed on resource-constrained devices.

We expect that privacy and security issues related to NVS would attract increasing attentions and interests from both academia and industry in the upcoming years, given the promising advantages and applications of NVS. We will be continuously working in this emerging field, exploring more potential threats to NVS, and proposing novel approaches and solutions to meet the challenges.

The following abbreviations are used in this manuscript:

## Figures and Tables

**Table 1:** The PSNR and SSIM results of the comparison between the original reconstructed images and the reconstructed images after using different encryption algorithms.

Dataset	Sequence	Ours		95% Discarding		95% Scrambling	
		PSNR	SSIM	PSNR	SSIM	PSNR	SSIM
**DAVIS**	dynamic_6dof	**5.43**	**0.07**	14.60	0.53	13.47	0.13
	poster_6dof	**7.27**	**0.06**	15.26	0.21	14.19	0.10
	shapes_rotation	**5.98**	**0.11**	14.86	0.79	8.16	0.15
	outdoors_running	11.77	**0.09**	12.06	0.40	**10.82**	0.16
**DDD17**	rec1487779465	**5.17**	**0.07**	13.32	0.61	9.19	0.13
	rec1487839456	**4.02**	**0.03**	14.84	0.53	4.47	0.06
	rec1487609463	**3.97**	**0.07**	17.49	0.83	14.04	0.15

**Table 2:** The UACI and NPCR results of the comparison between the original reconstructed images and the reconstructed images after using different encryption algorithms.

Dataset	Sequence	Ours		95% Discarding		95% Scrambling	
		UACI	NPCR	UACI	NPCR	UACI	NPCR
**DAVIS**	dynamic_6dof	**52.00**	**99.96**	15.10	98.94	18.78	99.55
	poster_6dof	**41.43**	**99.89**	14.19	99.13	16.07	99.25
	shapes_rotation	**50.03**	**99.99**	16.34	99.49	39.30	99.97
	outdoors_running	22.54	99.34	23.15	99.35	**25.22**	**99.56**
**DDD17**	rec1487779465	**54.35**	**99.95**	18.91	98.99	31.76	99.74
	rec1487839456	**61.35**	**99.99**	14.81	99.07	58.32	**99.99**
	rec1487609463	**64.30**	**99.99**	10.55	97.82	16.90	99.23

**Table 3:** Time (μs) spent on encrypting one event on Raspberry Pi (*K* = 36,694,061) using different updating scores and NVSs with different resolutions.

Dataset	Sequence	Events/s	Updating Score				
			7.2	7.6	8.0	8.4	8.8
Gait 128 × 128	2-1	17,034	0.549	0.261	0.114	0.179	0.115
	2-2	14,036	0.513	0.242	0.177	0.115	0.104
	2-3	11,545	0.535	0.252	0.183	0.110	0.111
DAVIS 240 × 180	shape_6dof	299,375	0.463	0.200	0.113	0.055	0.039
	shapes_rotation	385,438	0.511	0.222	0.097	0.056	0.039
	shapes_translation	289,400	0.461	0.200	0.099	0.055	0.039
DDD17 346 × 260	rec1487609463	153,625	0.563	0.243	0.127	0.069	0.050
	rec1487779465	474,789	0.559	0.245	0.124	0.075	0.051
	rec1487839456	680,153	0.564	0.246	0.120	0.072	0.051

**Table 4:** Time (μs) spent on encrypting one event on desktop server (*K* = 253,725,220) using different updating scores and NVSs with different resolutions.

Dataset	Sequence	Events/s	Updating Score				
			7.2	7.6	8.0	8.4	8.8
Gait 128 × 128	2-1	17,034	0.619	0.180	0.086	0.039	0.024
	2-2	14,036	0.415	0.167	0.080	0.038	0.023
	2-3	11,545	0.418	0.175	0.084	0.041	0.023
DAVIS 240 × 180	shape_6dof	29,9375	0.398	0.163	0.068	0.031	0.016
	shapes_rotation	385,438	0.398	0.163	0.069	0.032	0.016
	shapes_translation	289,400	0.398	0.163	0.068	0.031	0.016
DDD17 346 × 260	rec1487609463	153,625	0.419	0.171	0.072	0.033	0.017
	rec1487779465	474,789	0.418	0.171	0.072	0.033	0.018
	rec1487839456	680,153	0.420	0.171	0.073	0.035	0.018

**Table 5:** Time (μs) spent on encrypting one event on cloud server (*K* = 152,031,121) using different updating scores and NVSs with different resolutions.

Dataset	Sequence	Events/s	Updating Score				
			7.2	7.6	8.0	8.4	8.8
Gait 128 × 128	2-1	17,034	0.406	0.173	0.069	0.034	0.023
	2-2	14,036	0.413	0.170	0.075	0.032	0.023
	2-3	11,545	0.416	0.169	0.068	0.032	0.024
DAVIS 240 × 180	shape_6dof	299375	0.400	0.161	0.067	0.029	0.014
	shapes_rotation	385,438	0.399	0.161	0.067	0.029	0.014
	shapes_translation	289,400	0.400	0.162	0.067	0.029	0.014
DDD17 346 × 260	rec1487609463	153625	0.406	0.165	0.069	0.030	0.015
	rec1487779465	474,789	0.406	0.166	0.069	0.030	0.015
	rec1487839456	680,153	0.411	0.166	0.069	0.031	0.015

## Data Availability

The data invovled in this study can be access from: DAVIS dataset: http://rpg.ifi.uzh.ch/davis_data.html (accessed on 25 January 2021), DAVIS driving dataset (DDD17) http://sensors.ini.uzh.ch/databases.html (accessed on 25 January 2021) and Gait dataset (EV Gait): https://github.com/zhangxiann/TPAMI_Gait_Identification (accessed on 10 May 2021).
